# Foetal ductus arteriosus constriction unrelated to non-steroidal anti-Inflammatory drugs: a case report and literature review

**DOI:** 10.1080/07853890.2021.1921253

**Published:** 2021-06-07

**Authors:** Giovanna Battistoni, Ramona Montironi, Jacopo Di Giuseppe, Luca Giannella, Giovanni Delli Carpini, Alessandra Baldinelli, Marco Pozzi, Andrea Ciavattini

**Affiliations:** a Woman’s Health Sciences Department, Gynecologic Section, Polytechnic University of Marche, Ancona, Italy; b Department of Paediatric and Congenital Cardiac Surgery and Cardiology, Ospedali Riuniti, Ancona, Italy

**Keywords:** Ductus arteriosus constriction, NSAID, foetal, solvents, maternal exposure

## Abstract

Foetal ductus arteriosus (DA) constriction can be found in complex foetal heart malformations, but rarely as an isolated defect. Although many cases of DA constriction are usually related to Non-steroidal Anti-Inflammatory Drugs (NSAIDs) maternal intake, other causes remain without an established aetiology and are referred to as idiopathic. Recently, a wide range of risks factors or substances (polyphenol-rich foods intake, naphazoline, fluoxetine, caffeine and pesticides) showed a definitive effect upon the pathway of inflammation, causing DA constriction. We report a case of a premature DA constriction in a woman whose possible risk factor was identified in her maternal occupational exposure to solvents and a comprehensive literature review of 176 cases of NSAID-unrelated DA constriction. A 30-year-old Asian woman was referred to our institution at 33 gestational weeks and 0 days because of suspicion of premature DA constriction. The woman had no history of medication intake, including NSAIDs, alcohol, tobacco or polyphenol-rich-food consumption during pregnancy. A detailed foetal echocardiography revealed a normal cardiac anatomy with hypertrophic, hypokinetic and a dilated right ventricle due to right pressure overload, holosystolic tricuspid regurgitation, and, at the level of the DA, high systolic and diastolic velocities, indicating premature ductal restriction. The right outflow showed dilatation of the pulmonary artery with narrow DA. An urgent caesarean section was performed at 33 gestational weeks and 4 days due to worsening of DA PI and signs of right pressure overload, despite the interruption of exposure to solvents. We assume a relationship exists between premature DA constriction and a maternal occupational exposure to solvents. This hypothesis is reinforced by the presence of associated foetal malformations in in two of the patient’s children. Further research is needed to confirm the role of exposure to solvents and toxic chemicals in the pathogenesis of DA constriction, also with experimental animal models.

**KEY MESSAGES**
1. Many cases of DA constriction are usually related to Nonsteroidal Anti-Inflammatory Drugs (NSAIDs) maternal intake.
2. A wide range of risks factors or substances (polyphenol-rich foods intake, naphazoline, fluoxetine, caffeine and pesticides) can cause foetal DA constriction.
3. Further investigation are needed to confirm the role of maternal exposure to solvents in the pathogenesis of DA constriction.

## Introduction

The ductus arteriosus (DA) is an essential component of foetal circulation. Connecting the pulmonary artery to the descending aorta, it allows 80–85% of the right ventricle output to reach the systemic circulation, bypassing the high resistance fluid-filled lungs [1–4]. This communication between the pulmonary and systemic circulations establishes the parallel circulation in the foetus and equalizes pressure in the right and left ventricle. The patency of DA is maintained during gestation by locally produced and circulating prostaglandins, especially Prostaglandin E2 (PGE2), nitric oxide and low foetal oxygen saturation [5].

With advancing gestation, the DA becomes more sensitive to constricting factors, because it is subject to a progressive vascular remodelling to prepare itself to postnatal closure [6]. This histological maturation process starts at the second trimester and consists of the thickening of muscular layer [7].

Premature intra-uterine DA constriction could be diagnosed in complex congenital heart malformations, including Tetralogy of Fallot and truncus arteriosus. As an isolated defect, it is usually secondary to the use of medication like NSAIDs, isoxsuprine, fluoxetine and some foods rich in polyphenol like herbal teas, dark chocolate, orange juice, red/purple grapes, berries and coffee [8–12].

The mechanism of NSAID action is inhibition of prostaglandin production by direct constriction of the enzyme cyclooxygenase (COX). Production of prostaglandins is dependent on two enzymes which act in different states, cyclo-oxygenase-1 (COX-1), expressed endogenously, and cyclo-oxygenase-2 (COX-2), locally induced during the inflammatory processes [13]. Both animal and human studies have demonstrated constriction of the ductus after administration of prostaglandin synthetase inhibitors. This effect was not shown to depend on foetal serum concentration of the drug [14,15]. In recent years, also the antiinflammatory and antioxidant effects of foods rich in polyphenol have been demonstrated [16]; these effects are secondary to inhibition of the metabolic route of prostaglandins, especially of COX-2, preventing the transformation of arachidonic acid into prostaglandin [9].

Other possible risk factors could be the exposure to solvents or chemicals, but more case confirmations are required [17–18]. Idiopathic premature ductal constriction is considered a rare event.

We describe the case of a premature DA constriction in a woman whose possible risk factor was identified in her maternal occupational exposure to solvents. Moreover, we report, for the first time, a literature review on all cases of DA constriction unrelated to NSAID or congenital heart defects, to investigate the role of others risk factors.

## Case presentation

**Figure 1.**
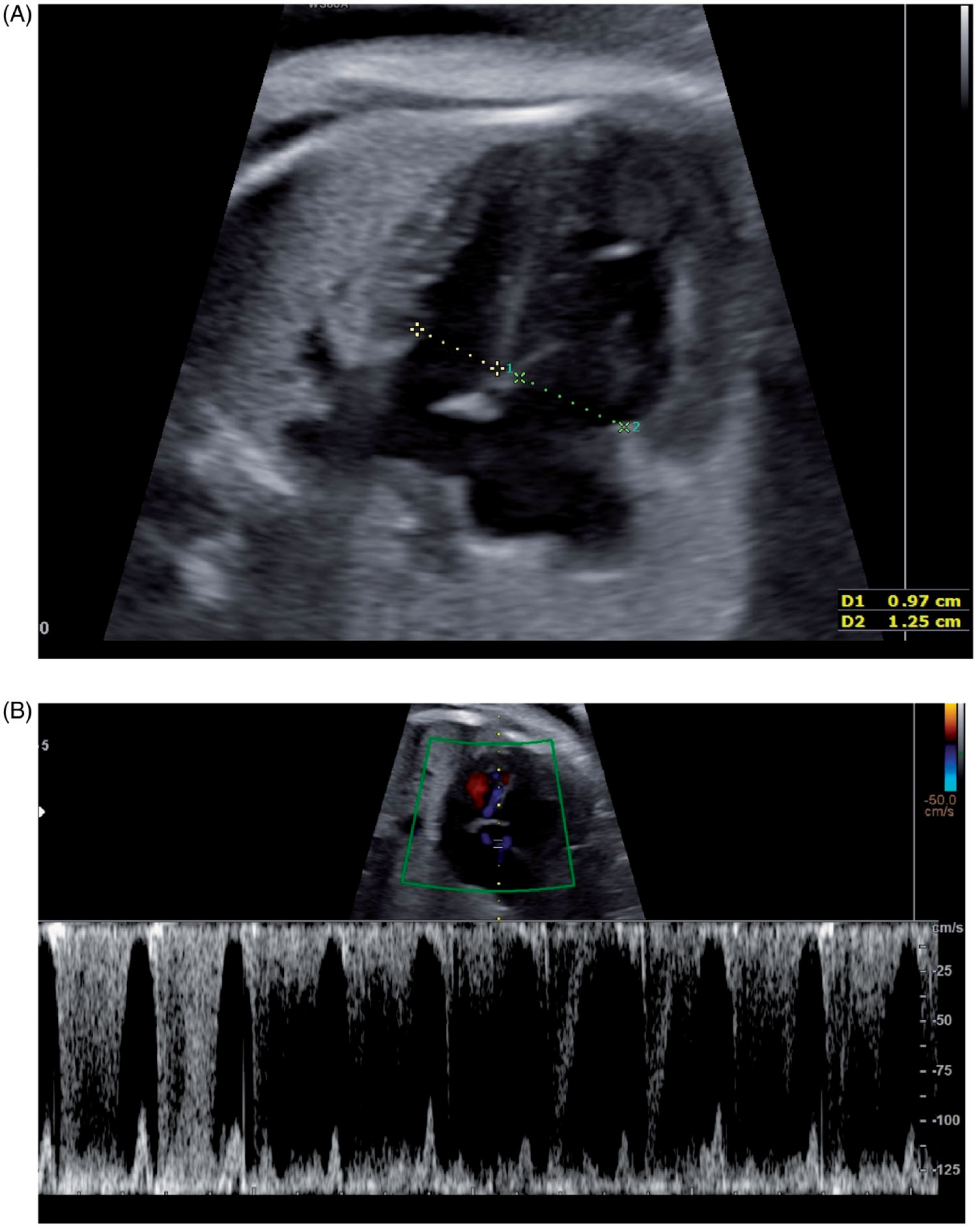
(A) Four chamber view at 33 gestational weeks: hypertrophic and dilated right ventricle, with mild pericardial effusion. (B) Tricuspid valve regurgitation peak velocity (130 cm/s).

**Figure 2.**
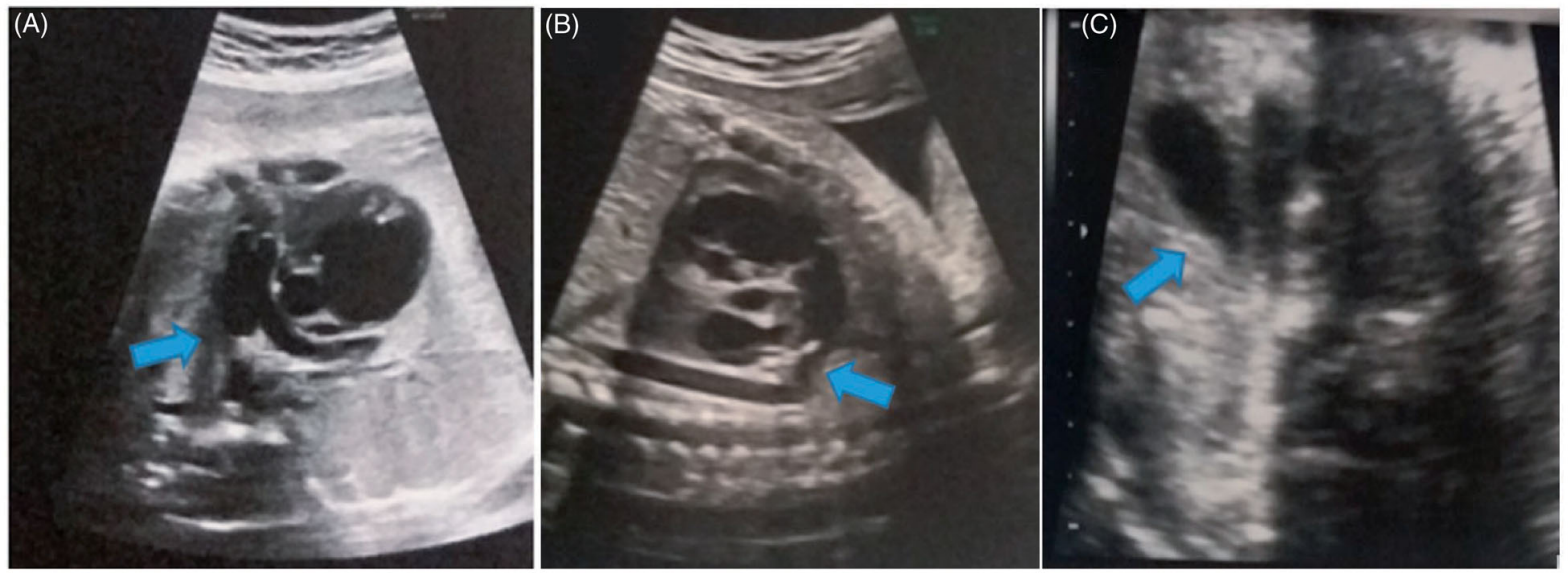
Two-dimensional echocardiography, showing ductus arteriosus constriction (arrow). (A) Right outflow tract. (B) Ductal arch view. (C) three-vessel view.

A 30-year-old Asian woman was referred to our institution at 33 gestational weeks and 0 days because of a suspicion of premature DA constriction on a routine third trimester ultrasound. The patient signed a standard written informed consent form for the use of data, pictures, and videos used for teaching and research purposes. This was the third pregnancy. The first newborn was affected by a lip and palate cleft, while the second one was healthy. The current pregnancy had no complications. The woman had no history of medication intake, including NSAIDs, alcohol, tobacco or polyphenol-rich-food consumption during pregnancy. In particular, in order to quantify the polyphenol ingestion, a food frequency questionnaire for consumption of polyphenol-rich foods in pregnant women was performed [19–20]. The total dietary amount of flavonoids was calculated from the USDA Database for the Flavonoid Content of Selected Foods [21], considering the 27 items with higher concentrations of polyphenols higher than 30 mg/100 g of food (green and black tea, mate tea, grape derivatives, dark chocolate, orange juice, fruit teas, olive oil, soy beans, berries, tomato, apples, spinach, and others) as reported by Zielinsky et al. [22]. On the other hand, her occupational exposure to solvents and toxic chemicals, as a hairdresser, (especially cosmetic products) resulted from the maternal and paternal history. A detailed foetal echocardiography revealed a normal cardiac anatomy with hypertrophic, hypokinetic and dilated right ventricle due to right pressure overload. The effects of premature DA constriction (mild pericardial effusion and a dilated and poorly functioning right ventricle) can be seen in Figure 1. The colour and pulsed Doppler interrogation showed holosystolic tricuspid regurgitation (130 cm/s) (Figure 1(B)) with jet that reached the roof of the atrium and at the level of the DA showed high systolic (200 cm/s) and diastolic (80 cm/s) velocities with a reduction in the pulsatility index (PI) (0.8), indicating premature ductal restriction. The right outflow showed dilatation of the pulmonary artery with narrow DA (Figure 2(B)). After the administration of corticosteroids, an urgent caesarean section was performed at 33 gestational weeks and 4 days due to worsening of DA PI and signs of right pressure overload, despite the interruption of exposure to solvents. A 2250 g-male neonate born with Apgar score of 5 and 9 at 1 and 5 min respectively. Post-natal echocardiography revealed an anatomically normal heart with progressive improvement of hypertrophy and right ventricular dilatation.

The newborn was treated immediately after birth with PGE infusion with the aim of reducing the pressure overload of the right ventricle and pulmonary hypertension. This use of prostaglandins is off-label, but free from major side effects. Due to poor response to PGE treatment, it was stopped after 18 h, and therapy with inotropic agents (dopamine) and nitric oxide was initiated to reduce the pulmonary pressure. Closure of DA took place 30 h after birth. Collaterally, congenital cataract was found. Normal human karyotype was found in the newborn.

## Methods: comprehensive review of the literature

**Figure 3.**
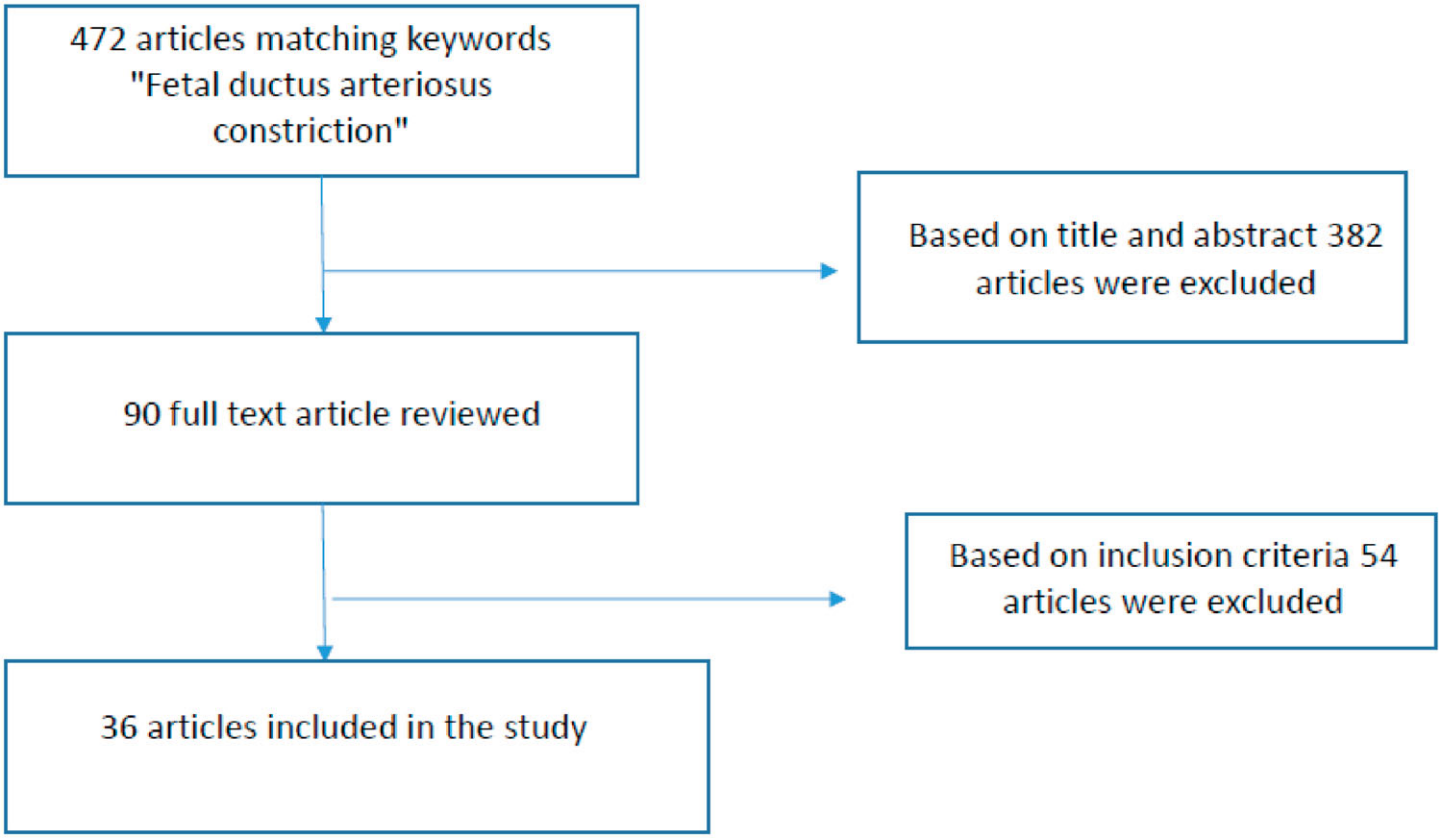
Search strategy flowchart.

The electronic medical database Medline/PubMed was used for research, combining the following terms: foetal ductus arteriosus constriction (472 articles). Titles and abstracts of these articles were screened for relevance by authors to determine which articles were to undergo full-text review (human cases of prenatal DA constriction/closure no NSAIDs or CHD induced). Animal cases of prenatal ductus arteriosus constriction, cases of NSAID related DA constriction, or related to heart defects were excluded (Figure 3). Articles identified at this stage as potentially relevant moved into full text review (Figure 3). The bibliographies of included studies were reviewed to identify additional publications not found through the database search.

## Results

To date, 176 cases of NSAID-unrelated (and congenital hearth defects- unrelated, CHD) premature DA constriction have been reported in the English language literature (from 1946 to 2020).

Including the present report, there are 177 cases of NSAID-unrelated (and not related to hearth disease) [4,8,23–54] (Table 1).

**Table 1.** Prenatal restriction/closure of DA: human cases in literature no NSAIDs or CHD induced (1946–2020).

Authors (Y)	Sample size	N	Study design	MA (y)	Causative agent (Substance exposure/idiopathic)	Dominant echo findings	GA at diagnosis (W)	Treatment	Delivery	GA at birth (w)	Postnatal presentation	Postnatal treatment and course
Battistoni et al. (the present report)	1	1	Case report	30	Solvents and toxic chemicals	RV dilatation, PA dilatation, narrowed DA, ↓ PI on DA, TR, pericardial effusion.	33	CS after corticosteroids	Immediate CS after corticosteroids	34	Progressive improvement of hypertrophy and right ventricular dilatation	Uneventful. Congenital cataract was found
Enzesberger et al. 2012 [23]	3	2	Case series	29	Idiopathic	TR, RA dilatation, constricted DA, ↑ PSV,PDV and ↓ PI on DA	33	Daily FU	CS (maternal request)	38	A, Normal-sized heart	Discharged d 4
		3		29	Idiopathic	RV dilatation, ↑ PSV,PDV and ↓ PI on DA, negative a wave DV	34	Daily FU	CS (breech- ↓RH function	35	A, Hypertrophic RV	Discharged d 16
		4		26	Idiopathic	Cardiomegaly, RH hypertrophy, TR, ↑ PSV,PDV and ↓ PI on DA, negative a wave DV	36	CS	CS (non-reassuring stress test)	36	A, RV hypertrophy, TR	Discharged d 5
Genovese et al.2015 [4]	1	5	Case report	38	Paracetamol	RV hypertrophy, ↑ PSV,PDV and ↓ PI on DA, tortuous S-shaped DA	34	CS after corticosteroids	Elective CS	35	RDS, Marked RV hypertrophy with impaired function, little and tortuous DA	Oxygen by nasal cannula Discharged d 15
Lopes et al 2015 [8]	16	6	Retrospective analysis	16–43	Idiopathic	RV dilatation, severe TR, ↑ PSV,PDV and ↓ PI on DA	29	FU	ND	ND	A	Uneventful
		7		16–43	Idiopathic	↑ PSV,PDV and ↓ PI on DA	34	FU	ND	ND	A	Uneventful
		8		16–43	Idiopathic	RV dilatation, mild TR↑ PSV,PDV and ↓ PI on DA	32	FU	ND	37	A	Uneventful
		9		16–43	Idiopathic	RV dilatation and akinetic, severe TR, pericardial effusion, ↑ PSV,PDV and ↓ PI on DA	37	Immediate delivery	ND	ND	Severe PH, severe RV dysfunction	Normal heart 3m
		10		16–43	Idiopathic	RV dilatation, mild TR, ↑ PSV,PDV and ↓ PI on DA	34	FU	ND	ND	A	Uneventful
		11		16–43	Idiopathic	RV dilatation, severe TR, ↑ PSV,PDV and ↓ PI on DA	36	FU	ND	ND	A	Uneventful
		12		16–43	Idiopathic	RV dilatation, mild TR, ↑ PSV,PDV and ↓ PI on DA	35	FU	ND	ND	A	Uneventful
		13		16–43	Idiopathic	RV dilatation, mild TR, ↑ PSV,PDV and ↓ PI on DA	28	FU	ND	ND	A	Uneventful
		14		16–43	Naphazoline (abusive use of nasal drops)	Mild TR, ↑ PSV,PDV and ↓ PI on DA	38	FU	ND	ND	A	Uneventful
		15		16–43	Asthma attack after pest control (unknown pesticide) with bronchodilators	RV dilatation, severe TR, ↑ PSV,PDV and ↓ PI on DA	33	FU	CS for persistent DA constriction	37	PH	Normal heart 15 d
		16		16–43	Isoxsuprine-B2 agonist	RV dilatation, moderate TR, ↑ PSV,PDV and ↓ PI on DA	34	FU	ND	ND	A	Uneventful
		17		16–43	Caffeine (abusive ingestion of cola soft drink, 3–4 l/d)	RV dilatation, moderate TR, ↑ PSV,PDV and ↓ PI on DA	31	FU	ND	ND	A	Uneventful
		18		16–43	Fluoxetine 60 mg/d (since beginning of pregnancy)	↑ PSV,PDV and ↓ PI on DA,	28	FU	ND	ND	A	Uneventful
		19		16–43	Caffeine (abusive ingestion of cola soft drink)	↑ PSV,PDV and ↓ PI on DA	33	FU	ND	ND	A	Uneventful
		20		16–43	Oxymetazoline+ Naphazoline (abusive use of nasal drops)	RV dilatation, mild TR, ↑ PSV,PDV and ↓ PI on DA C	34	FU	ND	ND	A	Uneventful
		21		16–43	Caffeine (abusive ingestion of cola soft drink)	RV dilatation, ↑ PSV,PDV and ↓ PI on DA	30	FU	ND	ND	A	Uneventful
Trevett et al. 2004 [24]	1	22	Case report	34	Idiopathic	Moderate RV hypertrophy, mild TR, ↑ PSV and ↓ PI on DA, tortuous S-shaped DA	33	Weekly FU	Induction for GDM, VD	38	A, hypertrophic RV	Discharged d 3
Rakha S. 2017 [10]	1	23	Case report	23	Orange intake (up to 2 kg/d)	RH dilatation, ↑ PSV,PDV and ↓ PI on DA, narrowed DA	31	Stop orange intake + FU	Spontaneous VD	39	A, Normal heart	Uneventful
Okada et al 2018 [25]	1	24	Case report	27	Idiopathic	LV and RA dilatation, severe TR, hypertrophic RV, narrowed DA, no blood flow on DA	37	CS	Emergency CS (sinusoidal pattern on CTG)	37	Severe dyspnoea, dilated cardiomyopathy	Respiratory support (intubation), Inotropes and diuretic administration.
												Discharged d 47.
												Resolution of cardiomyopathy 6 m
Shima et al.2010 [26]	1	25	Case report	27	Idiopathic	RA dilatation, severe TR, hypertrophic RV, narrowed DA, pericardial effusion	38	CS	Emergency CS	38	Tachypnea,RA dilatation, massive TR, hypertrophic RV, mild pericardial effusion,	Oxygen
												Discharged d 7
												Normal heart 3 m
Vian et al. 2018 [27]	35	26–60	Case-control	ND	Idiopathic	↑ PSV,PDV and ↓ PI on DA, narrowed DA,TR	≥28	Polyphenol-rich food restriction	ND	ND	A, Normal-sized heart	Uneventful
Yaman et al. 1999 [28]	1	61	Case report	ND	Idiopathic	RV hypertrophy , PA retrograde flow, ↑ PSV,PDV and ↓ PI on DA	39	ND	ND	39	PH	ND
Azancot-Benisty et al. 1995 [29]	1	62	Case report	38	Betamethasone (four courses )	RV hypertrophy, ↑ PSV,PDV and ↓ PI on DA, TR, mild pericardial effusion, narrowed DA	27	Stop steroids	CS for placenta Previa	38	A, normal-sized heart	Uneventful
Wei S. et al 2011 [30]	1	63	Case report	28	Idiopathic	No flow through DA, no narrowing of DA, RH dilatation, RV hypertrophy, severe TR, negative a wave on DV	38	CS	Emergency CS	38	A, mild TR, moderate PH, cardiomegaly, RV hypertrophy, closed DA	Uneventful
												Discharged d 3
												Normal heart d 14
Inatomi et al. 2017 [31]	1	64	Case report	38	Idiopathic	Cardiomegaly, dilatation of pulmonary trunk, ↑ PSV,PDV and ↓ PI on DA, moderate TR, narrowed DA	36	CS	Emergency CS (progression to hydrops)	36	dyspnoea, PH, severe TR with rupture of the anterior papillary muscle, RV hypertrophy	Oxygen, CPAP, cardiotonic drugs, NO (until d 7)
Sridharan et al. 2009 [32]	2	65	Case report	34	Camomile tea	↑ PSV,PDV and ↓ PI on DA, narrowed DA	20	Stop tea	ND	ND	ND	ND
		66		32	Camomile tea	RV dilatation and poorly contractile, moderate TR and PR, ↑ PSV,PDV and ↓ PI on DA, narrowed DA	35	CS	Immediate CS	35	A, DA closed, dilatated RV, mild TR, PR	Uneventful
Hayes 2016 [33]	1	67	Case report	33	Bio-Oil® (x2/d from II trim)	RA dilatation, hypertrophic and poorly contractile RV, moderate TR, pericardial effusion,↑ PSV,PDV and ↓ PI on DA, narrowed DA, negative a wave on DV	37	CS	Immediate CS	37	Dyspnoea, cardiomegaly, PH, RV systolic dysfunction, TR	Oxygen
												Discharged d 6.
												Normal heart 6 m
Srinivasan et al 2018 [34]	4	68–71	Case series	20–34	ALGS/WS	RV hypertrophy and dilatation, ↑ PSV,PDV and ↓ PI on DA, TR, narrowed DA	21–36	Follow up	Induction/CS (non-reassuring CTG)	32–36	Dyspnoea, PH, RV hypertrophy, ↑ flow velocities on peripheral PA	Oxygen up to 6 m
												Normal heart 6 m, bur PPS persisted.
Schierz et al. 2018 [35]	1	72	Case report	ND	Paracetamol (3g/d four 4 d in the III trimester), polyphenol rich-foods	ND	38	CS	Emergency CS	38	RDS, closed DA, severe cardiomyopathy, RV dysfunction, functional PA stenosis	Oxygen up to 6 d.
												Cardiomyopathy regression at 2 m.
Hofstadler et al. 1995 [36]	4	73	Case report	ND	Idiopathic	RV hypertrophy and dysfunction, TR, PR	37	Induction	CS	37	Dyspnoea, PH, closed DA, RV hypertrophy and dilatation	Oxygen for 46h.
												Discharged d 9.
												Normal heart at 7 w.
		74		ND	Idiopathic	RV hypertrophy and dysfunction, TR, abnormal umbilical vein pulsations, PA regurgitation	37	Induction of labour	VD	37	Dyspnoea, closed DA, RV hypertrophy and dilatation. Hyperechoic RV endocardium and papillary muscle.	Oxygen for 36h
												Antibiotics (sepsis).
												Discharged d 9.
		75		ND	6-days course antibiotics and phenyldimethylpyrazolam, glucocorticoids and ß-blocker	Closed DA, RV hypertrophy and dysfunction, ascites, TR, abnormal umbilical vein pulsations, PA regurgitation	38	CS	Emergency CS	38	Dyspnoea, PH, closed DA, RV hypertrophy and dilatation ,TR	Oxygen for 14h.
												Discharged d 6.
												At 3 m uncomplete regression of RV hypertrophy, but baby is clinically well.
		76		ND	Bethametasone single course	RV hypertrophy and dysfunction, TR, PA regurgitation				34	Induction of labour	VD	35	Closed DA, RV Hypertrophy and dilatation, mild TR	Discharged d 8.
							At 5w uncomplete regression of RV hypertrophy, but baby asymptomatic.								
Soslow et al. 2008 [37]	1	77	Case report	ND	Bethametasone single course	Resctricted DA.	31	Weekly FU	Emergency CS (worsening of RV function)	32	Closed DA, RV hypertrophy and dilatation, mild TR	Normal RV function, with mild residual RV hypertrophy at 3 w			
						RV hypertrophy and dysfunction, TR.									
						Abdominal meconium pseudocyst.									
Choi et al. 2013 [38]	1	78	Case report	22	Idiopathic	RV hypertrophy, RA dilatation, tortuous S-shaped DA, no flow on DA, mild TR	33	Induction	VD	34	Dyspnoea, closed DA, RV hypertrophy, mild TR	Oxygen with mechanical ventilator.			
												Discharged d 12			
												Normal heart 7 m.			
Zielinsky et al. 2012 [39]	51	79–129	Case-control	28 ± 6.5	Polyphenol rich-foods	↑ PSV,PDV and ↓ PI on DA, turbulent flow on DA, TR, RV hypertrophy	32 ± 3	Polyphenol-rich food restriction and FU after 3w	Spontaneous delivery	ND	A, normal sized heart	Uneventful			
Mielke et al. 1995 [40]	1	130	Case report	28	Idiopathic	↑ PSV,PDV and ↓ PI on DA, S-shaped DA, severe TR, RA and RV dilatation, transient PR	32	FU	CS (↑ tricuspid valve insufficiency)	36	Closed DA, RV hypertrophy and dilatation, mild TR	Progressive normal heart in the following d.			
Ishida et al. 2011 [41]	1	131	Case report	29	Idiopathic	↑ PSV,PDV and ↓ PI on DA, mild TR, RH dilatation, PR, hydrops	32	CS	Emergency CS	32	closed DA, dyspnoea, RV hypertrophy and dilatation, mild TR	Oxygen Endotracheal intubation, Catecholamine, Discharged d 31. Normal heart 2 m.			
Mielke et al. 1996 [42]	1	132	Case report	34	Idiopathic	↑ PSV and ↓ PI on DA, narrowed DA, RA dilatation, foetal atrial flutter	31	Weekly FU, digoxin + verapamil to obtain cardioversion	Spontaneous	39	RV hypertrophy. RA dilatation	Normal heart 3 m			
Gewillig et al. 2017 [43]	19	133	Case series	ND	Idiopathic	↑ PSV and PDV, ↓ PI on DA, narrowed DA, severe RV dilatation and hypertrophy	27	FU	Spontaneous	40	A, severe RV hypertrophy	Resolved			
		134		ND	Idiopathic	↑ PSV and PDV, ↓ PI on DA, narrowed DA, severe RV dilatation	26	FU, Induction (↑ RH dysfunction)	VD	36	Cyanosis,, severe RV dilatation and hypertrophy	CPAP			
		135		ND	Idiopathic	↑ PSV, and PDV, ↓ PI on DA, narrowed DA, severe RV hypertrophy	28	FU, Induction (progression RH dysfunction)	VD	38	A, severe RV hypertrophy, critical Pulmonary stenosis	Pulmonary atresia angioplasty, stent DA			
		136		ND	Paracetamol	↑ PSV, and PDV, ↓ PI on DA, narrowed DA, Pulmonary atresia dilatation	24	FU	Spontaneous VD	40	A, Pulmonary stenosis	Pulmonary atresia angioplasty			
		137		ND	Paracetamol	↑ PSV, and PDV, ↓ PI on DA, narrowed DA, severe TR, severe RV dilatation, pericardial effusion	25	FU, Induction (progression RH dysfunction)	VD	37	Cyanosis, PH, severe TR, moderate RV dilatation, severe RH dilatation	IPPV, NO, Inotropes, Tricuspid valve repair at 3 w			
		138		ND	Idiopathic	↑ PSV, and PDV, ↓ PI on DA, narrowed DA, severe TR, severe RH dilatation	37	FU	CS	39	Cyanosis, SVT, mild TR, severe RV hypertrophy, RA dilatation	Oxygen, Ablation 2 m			
		139		ND	Idiopathic	↑ PSV, and PDV, ↓ PI on DA, narrowed DA, severe RV hypertrophy	32	FU	Spontaneous VD	40	A, moderate RV hypertrophy	Resolved			
		140		ND	Idiopathic	↑ PSV, and PDV, ↓ PI on DA, no flow DA, severe RV hypertrophy	34	FU	VD	40	Cyanosis, sever PH, severe TR, severe RV hypertrophy, RVOTO	IPPV, NO, inotropes, death 3 m after attempted palliative surgery of RVOTO			
		141		ND	Idiopathic	↑ PSV, and PDV, ↓ PI on DA, no flow DA, severe TR, moderate RV dilatation	27	FU, Induction (↑RH dysfunction with hydrops)	VD	29	Cyanosis, PH, severe RV hypertrophy, cardiomyopathy	IPPV, NO, inotropes, mitral valve ring a 4 y			
		142		ND	Idiopathic	↑ PSV, and PDV, ↓ PI on DA, no flow DA, severe TR, moderate RH dilatation, severe RV hypertrophy	34	FU, Induction for progression RH dysfunction	VD	35	Cyanosis, PH, severe TR, moderate RH dilatation, severe RV hypertrophy, functional PuV atresia	IPPV, NO, inotropes, death on day 1 due to high pulmonary vascular resistance			
		143		ND	Idiopathic	↑ PSV, and PDV, ↓ PI on DA, no flow DA, Moderate RV hypertrophy	33	FU, Induction (↑RH dysfunction)	VD	34	Cyanosis, mild TR, severe RV hypertrophy	CPAP, resolved			
		144		ND	Idiopathic	↑ PSV, and PDV, ↓ PI on DA, no flow DA, Moderate RV hypertrophy ad dilatation, mild TR, microcystic lungs	20	FU	Spontaneous VD	39	Cyanosis, Air trapping, mild RV dilatation, severe RV hypertrophy, aneurismal dilatation PA trunk and branches	IPPV, inotropes, death at 3 for respiratory failure			
		145		ND	Idiopathic	↑ PSV, and PDV, ↓ PI on DA, no flow DA, mild TR, Moderate RH dilatation, moderate RV hypertrophy, PS, PR	28	FU, Induction (↑RH dysfunction)	VD	37	Cyanosis, mild TR, Moderate RA dilatation, severe RV hypertrophy, PS/PR	CPAP, PuV replacement 2y and 8 y			
		146		ND	Paracetamol	↑ PSV, and PDV, ↓ PI on DA, no flow DA, mild RV dilatation, PuV thickened	21	FU	CS	39	Cyanosis, Moderate RV dilatation, Agenesis PuV	Oxygen, PuV replacement 7 days			
		147		ND	Idiopathic	↑ PSV, and PDV, ↓ PI on DA, no flow DA, Severe RV hypertrophy, Pericardial effusion, RV hypocontractilty	38	Immediate CS	CS	38	Cyanosis, severe RV hypertrophy	Oxygen, resolved			
		148		ND	Idiopathic	↑ PSV, and PDV, ↓ PI on DA, no flow DA, severe RV hypertrophy, severe TR, mild RA dilatation	33	FU	Spontaneous VD	39	A, severe RV hypetrophy	Resolved			
		149		ND	Idiopathic	↑ PSV, and PDV, ↓ PI on DA, no flow DA, severe TR, mild RH dilatation, Pericardial effusion	39	Induction	VD	39	A	Resolved			
		150		ND	Idiopathic	↑ PSV, and PDV, ↓ PI on DA, no flow DA, severe RV dilatation, mild RV hypertrophy	39	FU	Spontaneous VD	40	A, severe RV hypertrophy, mild TR	Resolved			
		151		ND	Idiopathic	↑ PSV, and PDV, ↓ PI on DA, no flow DA, mild RV dilatation, mild TR, severe RV hypertrophy	36	FU	Spontaneous VD	36	Cyanosis, mild RV hypertrophy	CPAP, resolved			
Babaoğlou et al. 2013 [44]	1	152	Case report	29	Idiopathic	↑ PSV, and PDV, ↓ PI on DA, narrowed DA, RH dilatation, RV Hypertrophy, mild TR, hydrops	33	CS	CS	33	Dyspnoea, closed DA, mild TR, mild RV hypertrophy	Oxygen. Discharged d 8. Normal heart d 8.			
Becker et al. 1977 [45]	2	153	Case report	ND	Idiopathic	ND	ND	None	Spontaneous VD	39	Cyanosis, asphyxia, hydrops, RH dilatation, markedly Narrowed DA	Death after delivery			
		154		ND	Idiopathic	ND	ND	None	Spontaneous VD	40	Asphyxia, hydrops, RA dilatation, markedly Narrowed DA	Death 1 h after delivery			
Leal et al. 1997 [46]	3	155	Case series	28–38	Idiopathic	No flow on DA, RV dilatation, mild TR, mild PuV insufficiency	32	ND	CS	ND	A, absent DA flow, RV dilatation	Uneventful, Normal-sized heart on follow up			
		156		28–38	Idiopathic	No flow on DA, RV dilatation, mild TR, mild PuV insufficiency	41	ND	CS	ND	A ,absent DA flow, RV dilatation	Uneventful, Normal-sized heart on follow up			
		157		28–38	Idiopathic	No flow on DA, RV dilatation, mild TR, mild PuV insufficiency	40	ND	CS	ND	A, absent DA flow, RV dilatation	Uneventful, Normal-sized heart on follow up			
Talemal et al. 2016 [47]	1	158	Case report	31	Dexamethasone (1w) for suspected myocardial inflammation in anti-SSA-exposed foetus	↑ PSV, and PDV, ↓ PI on DA, narrowed DA, mild RH dilatation, mild TR, hyperechoic Mitral valve	28	Follow up	Spontaneous VD	38	RDS, RH dilatation, RV dysfunction, no myocardial inflammation	Endotracheal intubation for 24h, normal heart at 2w.			
Eidem et al 2000 [48]	1	159	Case report	35	Idiopathic	narrowed DA	23	FU	Inducted VD for IUGR	38	A, constricted DA	Uneventful			
Corti et al. 2020 [49]	1	160	Case report	35	Sertraline (25mg/d) Lorazepam (10drops/d) Paracetamol (2–4 g/d first trimester and 1–2 g occasionally in the third trimester)	No flow on DA, severe RH dilatation, TR, PuV insufficiency, decreased function of RV, Negative a-wave on DV,				33	CS after single course of corticosteroids	CS	33	Dyspnoea, PH, No DA, RV hypertrophy and dilatation, mild PuV insufficiency	Oxygen by nasal cannula
							Normal heart 1 m.								
Kim et al. 2003 [50]	1	161	Case report	35	Idiopathic	↑ PSV, and PDV on DA, narrowed and S-shaped kinking DA, RH dilatation, RV Hypertrophy, mild TR, mild pericardial effusion	26	FU	CS (foetal distress)	31	Dyspnoea, PH, Tortuous DA, RV hypertrophy, mild TR, mild pericardial effusion	Oxygen with mechanical ventilator (1 w).			
												Discharged 5 w.			
												Normal heart 4 m.			
Ellis et al. 2013 [51]	1	162	Case report	ND	Lithium (throughout pregnancy)	RH dilatation	18	FU	Preterm delivery	ND	Closed DA	ND			
Becquet et al. 2018 [52]	1	163	Case series	27	Paracetamol (for 7 d after 34 w)	↑ PSV, PDV and ↓ PI on DA, narrowed DA, RV dilatation, mild TR	37	Induction	VD	37	A, mild TR, mild RV hypertrophy and hypocontractility, totally closed DA	Uneventful.			
												Progressive normal heart.			
												Discharged d 5.			
Chugh et al.2020 [53]	1	164	Case series	31	Idiopathic	↑ PSV, PDV and ↓ PI on DA, narrowed and S-shaped DA, mild TR	32	FU	CS (worsening RV dysfunction)	38	Dyspnoea, severe RV dilatation and hypertrophy, severe TR. Closed DA.	Mechanic ventilation for 2d.			
												Discharged d 10.			
												Normal heart at 1 m.			
Luchese et al. 2003 [54]	13	165	Retrospective analysis	19	Idiopathic	↑ PSV, PDV and ↓ PI on DA, RH dilatation, hypertrophic RV, mild PR	33	FU	ND	ND	PH	ND			
		166		32	Idiopathic	↑ PSV, PDV and ↓ PI on DA, dilatated/hypocontractile RV, mild TR	27	FU	ND	ND	PH	ND			
		167		17	Idiopathic	↑ PSV, PDV and ↓ PI on DA, dilatated RH and PA, mild TR	37	FU	ND	ND	A	Uneventful			
		168		35	Idiopathic	No flow on DA, dilatated RH and PA, severe TR and PR, hypertrophic RV	36	FU	ND	ND	ND	Uneventful			
		169		21	Idiopathic	↑ PSV and ↓ PI on DA, mild RV dilatation, mild TR	34	FU	ND	ND	ND	Uneventful			
		170		32	Idiopathic	↑ PSV, PDV and ↓ PI on DA, mild PR	31	FU	ND	ND	A	Uneventful			
		171		36	Idiopathic	↑ PSV, PDV and ↓ PI on DA, dilatated RH, mild PR	34	FU	ND	ND	A	Uneventful			
		172		25	Idiopathic	↑ PSV, PDV and ↓ PI on DA, dilatated/hypocontractile RH	32	FU	ND	ND	A	Uneventful			
		173		41	Idiopathic	↑ PSV, PDV and ↓ PI on DA, mild TR, dilatated RV, severe hydrops	28	FU	ND	ND	Neonate death	Neonate death			
		174		17	Idiopathic	↑ PSV, PDV and ↓ PI on DA	38	FU	ND	ND	A	Uneventful			
		175		20	Idiopathic	↑ PSV, PDV and ↓ PI on DA, mild TR	32	FU	ND	ND	A	Uneventful			
		176		28	Idiopathic	↑ PSV, PDV and ↓ PI on DA, dilatated RH, hypertrophic RV, mild PR	33	FU	ND	ND	A	Uneventful			
		177		39	Idiopathic	↑ PSV, PDV and ↓ PI on DA	33	FU	ND	ND	A	Uneventful			

GA: gestational age; W: weeks; Y: years; D: days; M: months; H: hours; FU: follow up; N: case number; MA: maternal age; RV: right ventricle; RA: right atrium; RH: right heart; PA: pulmonary artery; PuV: pulmonary valve; LF: left ventricle; PI: pulsatility index; DV: ductus venosus; PSV: peak systolic velocity; PDV: peak diastolic velocity; TR: tricuspid regurgitation; PR: pulmonary regurgitation; PS: pulmonary stenosis; RVOTO: right ventricle outflow tract obstruction; ND: no data available; CS: caesarean section; VD: vaginal delivery; PH: pulmonary hypertension; A: asymptomatic; CPAP: continuous positive airway pressure; IPPV: intermittent positive pressure ventilation; NO: nitrous oxide; NICU: neonatal intensive care unit; RDS: respiratory distress syndrome; SVT: supraventricular tachycardia; GDM: gestational diabetes; CTG: cardiotocography; ALGS: Alagille syndrome; WS: Williams syndrome; PPS: peripheral pulmonary stenosis.

**Figure 4.**
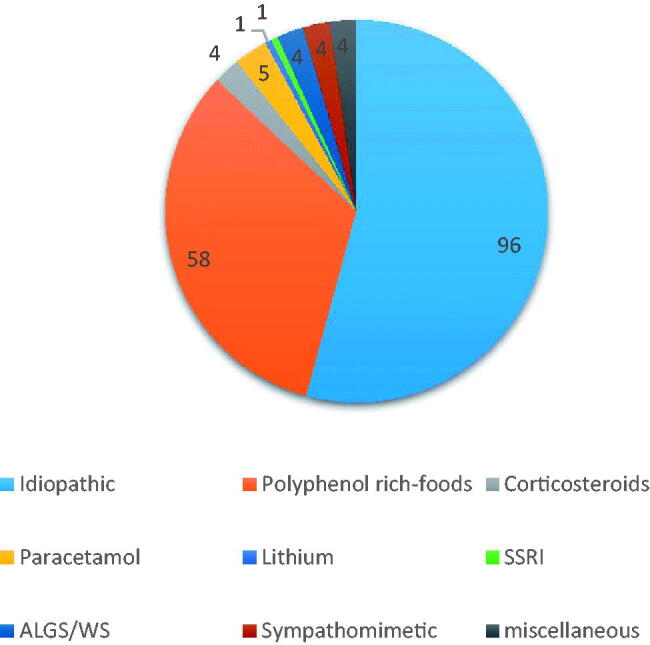
Distribution of etiopathogenesis of human cases in literature no NSAIDs or CHD induced.

Figure 4 report the distribution of etiopathogenesis of human cases in literature no NSAIDs or CHD induced; of the 177 cases found 96 were idiopathic (54.2%), 58 were related to polyphenol rich-food, 5 to paracetamol, 4 were related to genetic arteriopathy (Alagille and Williams Syndrome), 4 cases were related to sympatomimetics drugs, 4 to corticosteroids, 4 to miscellaneous causes, 1 to SSRI consumption and 1 case to lithium consumption. In the literature, many cases are considered as idiopathic, but no one reported about maternal employment. However, it would be important to investigate whether there is a common pathogenetic mechanism form in many cases, such as occupational exposure to solvents or intake of paracetamol (acetaminophen), a drug considered safe in pregnancy. In particular, a repeated dose intake, especially in the third trimester of pregnancy, can have a vasoconstrictive effect [55].

## Discussion

Patient history was accurately reviewed to identify a possible causative agent. The woman had no chronic illness and was not a smoker. The foetus’s heart had no congenital defects. We asked about medications (especially NSAIDs) and polyphenol-rich foods intake. The mother denied the consumption of any kind of medicine, herbal tea, grapes or other polyphenol-rich food during pregnancy. A dietary intervention for maternal restriction of polyphenol-rich foods or suspension of NSAIDs consumption in the third trimester of pregnancy is accompanied by increase in plasma levels of PGE2 and reversal of foetal ductal constriction [10,27,39,56,57].

In the absence of the most common aetiologies, the occupational exposure to solvents or an idiopathic premature constriction of DA was suggested. The occupation of both parents as hairdressers, which involved the daily use of organic solvents, could be suspected. Widely discussed in the literature is the association between maternal occupational exposure to solvents (as in hairdressing and cosmetology) and an increased risk of adverse obstetrics outcomes, such as spontaneous abortion, preterm birth, small for gestational age (SGA), low birth weight (LBW) and congenital malformations (especially cleft lip and palate, urinary malformations, hypospadias and eye diseases) [17,18,58–62]. Our case could underline this association. The mother did not stop working before and during pregnancy and the foetus had not only the premature DA constriction but also congenital cataract, without any other risk factors. In addition, the first child was affected by lip and palate cleft. Hairdressers are predominantly women, and many of them are of childbearing age. Hairdressers work in a complex environment where they are in daily contact with various chemical substances which can be found in hair care product used for washing, dyeing, bleaching, spraying and perming. Their main routes of exposure are dermal and respiratory. Several solvents have been shown to be teratogenic for animals. In mice, for example, toluene and xylene (petroleum solvents) have been associated with the occurrence of cleft palate [18], and ethylene glycol monomethyl ether has been associated with the occurrence of neural tube defects [62], while in zebrafish, p-phenylenediamine, often included in hair dye, it can cause cardiovascular defects [63]. Also aromatic amines and aldehydes could have a role in COX2 inhibition that determine congenital heart defects [64]. In humans, malformations and cytogenetic effects have been observed among the offspring of women exposed to glycol ethers during pregnancy [65]. Some studies [66–69], but not others [70], report an excess risk of spontaneous abortion among women occupationally exposed to solvents. A small prospective cohort [71], and a meta-analysis [72], performed by the same research group both report associations between maternal occupational exposure to solvents and major malformations. Two occupational cohort studies of women working in laboratories suggest similar results [73–74]. Various case-control studies have shown relations between maternal occupational exposure to solvents and some subtypes of malformations, mostly oral clefts [75–77]. Some significant associations have also been reported between maternal exposure to solvents and cardiac malformations [75,78], visual impairment [17] and neural tube defects [75,79].

## Conclusion

Premature constriction of DA is a rare event and in most cases is secondary to maternal intake of NSAIDs or foods rich in polyphenols. For the first time, the present review reported all cases of DA constriction not related to NSAIDs intake or to CHD.

The gynaecologist must take into account that there are not only forms of DA constriction secondary to the intake of NSAIDs. We assume a relationship between premature DA constriction and a maternal occupational exposure to solvents. This association between a maternal occupational exposure to solvents and an increased risk of adverse obstetrics outcomes has been widely discussed in the literature. In our case report and in the previous newborns this hypothesis is reinforced by the presence of other associated foetal malformations. It is therefore important to carry out through an occupational history and inform the patient about the potential risks associated with the exposure to solvents and toxic chemicals. Further investigation is needed to confirm their role in the pathogenesis of DA constriction, as in experimental animal models, such as those already performed in pregnant rats and sheep with polyphenols. A randomized clinical trial is needed to analyse the role of solvents in inducing this condition would be desirable, respecting the ethical aspects of the research.
